# Escin Activates Canonical Wnt/β-Catenin Signaling Pathway by Facilitating the Proteasomal Degradation of Glycogen Synthase Kinase-3β in Cultured Human Dermal Papilla Cells

**DOI:** 10.3390/cimb45070373

**Published:** 2023-07-14

**Authors:** Jae Young Shin, Jaeyoon Kim, Yun-Ho Choi, Sanghwa Lee, Nae-Gyu Kang

**Affiliations:** 1 LG Household & Health Care (LG H&H) R&D Center, 70, Magokjoongang 10-ro, Gangseo-gu, Seoul 07795, Republic of Korea; sjy2811@lghnh.com (J.Y.S.); kjy5281@lghnh.com (J.K.); youknow@lghnh.com (Y.-H.C.); 2 Innovo Therapeutics Inc., 507 38, Mapo-daero, Mapo-gu, Seoul 04174, Republic of Korea

**Keywords:** Escin, Wnt/β-catenin signaling, GSK-3β, post-translational modification, agonist

## Abstract

Abnormal inactivation of the Wnt/β-catenin signaling pathway is involved in skin diseases like androgenetic alopecia, vitiligo and canities, but small-molecule activators are rarely described. In this study, we investigated the stimulatory effects of escin on the canonical Wnt/β-catenin signaling pathway in cultured human dermal papilla cells (hDPCs). Escin stimulated Wnt/β-catenin signaling, resulting in increased β-catenin and lymphoid enhancer-binding factor 1 (LEF1), the accumulation of nuclear β-catenin and the enhanced expression of Wnt target genes in cultured hDPCs. Escin drastically reduced the protein level of glycogen synthase kinase (GSK)-3β, a key regulator of the Wnt/β-catenin signaling pathway, while the presence of the proteasome inhibitor MG-132 fully restored the GSK-3β protein level. The treatment of secreted frizzled-related proteins (sFRPs) 1 and 2 attenuated the activity of escin in Wnt reporter assays. Our data demonstrate that escin is a natural agonist of the canonical Wnt/β-catenin signaling pathway and downregulates GSK-3β protein expression by facilitating the proteasomal degradation of GSK-3β in cultured hDPCs. Our data suggest that escin likely stimulates Wnt signaling through direct interactions with frizzled receptors. This study underscores the therapeutic potential of escin for Wnt-related diseases such as androgenetic alopecia, vitiligo and canities.

## 1. Introduction

The Wnt/β-catenin signaling pathway plays a critical role in various cellular and physiological processes, such as cell proliferation, differentiation, survival, migration and polarity, during development and tissue homeostasis. Controlling the level of transcriptionally active β-catenin via the protein (β-catenin) destruction complex is central to the pathway since it determines the expression of Wnt target genes [1]. The β-catenin destruction complex consists of Axin, adenomatous polyposis coli (APC), GSK-3 and casein kinase 1(CK1) [2,3]. In the absence of Wnt, β-catenin is consecutively phosphorylated by CK1 and GSK-3, and the phosphorylated β-catenin is then ubiquitinated and consequently degraded by proteasomes [4,5,6]. On the other hand, when Wnt is present, the binding of Wnt with its receptor results in the dissociation of the destruction complex and the accumulation of β-catenin in the nucleus, where β-catenin binds to T-cell factors/lymphoid enhancer binding factors (TCFs/LEFs), triggering the expression of target genes [7,8].

**Figure 1:**
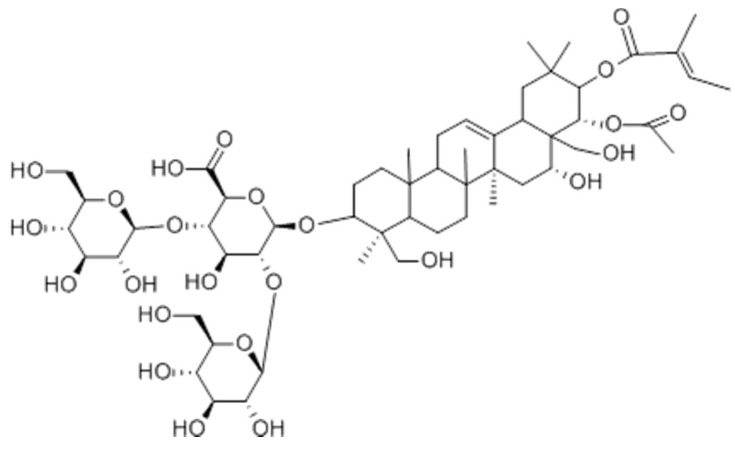
Chemical structure of escin.

As an essential regulator of tissue homeostasis and regeneration, Wnt/β-catenin signaling is tightly controlled in spatiotemporal patterns. Thus, dysregulation of the Wnt/β-catenin pathway is closely associated with many health conditions, including cancer, hair loss, pathological wound healing and osteoporosis [9,10,11,12]. The abnormal inactivation of this pathway is especially implicated in the pathogenesis of osteoporosis and skin-related diseases like androgenetic alopecia, premature hair greying and vitiligo, all of which severely compromise the quality of life of affected patients [13,14,15,16,17]. So, restoring the Wnt/β-catenin signaling pathway could be a promising strategy for treating and/or preventing these diseases, but commercially available small-molecule Wnt activators are rarely reported [18,19]. We screened hundreds of natural products in the WRHEK 293A Wnt reporter cell line and identified several chemicals that showed Wnt-stimulatory activity. Escin was selected as the first candidate because it is commercially available and has been widely used in pharmacologic and cosmetic products. Escin (Figure 1) is a mixture of pentacyclic triterpene saponins and is the major active component of horse chestnut seeds (*Aesculus hippocastanum*, L).

In this study, we examined the impact of escin on the Wnt/β-catenin signaling pathway in Wnt reporters and cultured human dermal papilla cells (hDPCs), and elucidated the underlying mechanisms of regulation. We found that escin is a canonical agonist of the Wnt/β-catenin signaling pathway. Our data demonstrate that escin stimulates Wnt signaling by facilitating the proteasomal degradation of GSK-3, possibly disrupting the β-catenin destruction complex in cultured hDPCs, and that the Wnt-stimulating activity of escin is likely mediated through the direct interaction with frizzled receptors. 

## 2. Materials and Methods

### 2.1. Materials

Escin and MG132 were purchased from Sigma-Aldrich (St. Louis, MO, USA). Recombinant Wnt3a and secreted frizzled related proteins (sFRPs) were purchased from R&D systems (Minneapolis, MN, USA).

### 2.2. Cell Culture 

The cell line of the Wnt reporter WRHEK 293A was purchased from AMSBIO (Cambridge, MA, USA). Cells were cultured in MEM (Corning, New York, NY, USA) supplemented with 10% FBS (Gibco BRL, Waltham, MA, USA), 100 U·ml^−1^ penicillin G and 100 µg·ml^−1^ streptomycin (Gibco BRL, Waltham, MA, USA). Human DPCs were purchased from Promocell (Heidelberg, Germany). The cells were cultured in basal medium supplemented with a supplement mix that contained 4% FBS, 0.4% bovine pituitary extract, 1 ng·ml^−1^ basic FGF and 5 μg·ml^−1^ insulin. Cells were maintained in a humidified incubator at 37 °C with 5% CO_2_.

### 2.3. TOPFlash Reporter Assay 

WRHEK293A cells (3 × 10^5^ cells per well) were seeded in black 96-well plates and cultured for 24 h. Cells were treated with various concentrations of escin and incubated for another 24 h. Cells were then lysed by adding 50 μL of 1× Passive Lysis Buffer (Promega, Madison, WI, USA) to each well and shaking for 10 min. GFP expression levels (internal cell viability control) were assessed by measuring the fluorescence at a 488/510 nm wavelength using VICTOR3 (PerkinElmer, Waltham, MA, USA). Then, 50 μL of luciferase substrate solution (Promega, Madison, WI, USA) was added and luciferase activity was measured using VICTOR3. Luminescence (TCF/LEF activity) values were normalized to GFP (cell viability) values.

### 2.4. Western Blotting

DPCs (1 × 10^6^ cells per dish) were seeded in 100 mm culture dishes and cultured for 24 h. Cells were treated with escin at various concentrations for 24 h, and then, washed with ice-cold PBS and lysed on ice in M-PER buffer (Thermo Fisher scientific, Waltham, MA, USA) supplemented with Complete^TM^ protease inhibitor cocktail and phosphatase inhibitor (Roche, Basel, Swiss). A total of 40 μg of protein was analyzed via Western blotting with antibodies to evaluate the following proteins’ expression levels: β-catenin (1000:1 dilution, Abcam, Cambridge, UK), GAPDH (1000:1 dilution, Santa Cruz, Dallas, TX, USA), LEF1, Axin2 (1000:1 dilution, Abcam, Cambridge, UK) and GSK-3β (1000:1 dilution, Cell signaling Technology, Danvers, MA, USA). Western blot was analyzed using a chemiluminescence detector (Vilber, Marne-la-vallée, France).

### 2.5. Quantitative Real-Time PCR

DPCs (1 × 10^6^ cells per well) were seeded in 6-well plates and cultured for 24 h. Then, the cells were treated with escin at various concentrations for 24 h. Total RNA was isolated using an RNA isolation kit (Qiagen, RNeasy mini kit) according to the manufacturer’s guide. cDNA was synthesized via reverse transcription using an eCube cDNA synthesis kit (philekorea, Seoul, Republic of Korea) with a PCR thermocycler (R&D systems, Minneapolis, MN, USA) according to the manufacturer’s protocol. cDNAs obtained from control cells and escin-treated cells were subjected to real-time PCR analysis. The TaqMan probes used in this study were as follows: GAPDH assay id 4352934E; CTNNB1 assay id Hs00355045_m1; LEF1 assay id Hs01547250_m1; Frizzled3 assay id Hs00907280_m1; Frizzled4 assay id Hs00201853_m1; Frizzled5 assay id Hs00258278_s1; Frizzled7 assay id Hs00275833_s1; CCND1 assay id Hs00765553_m1; Myc assay id Hs00153408_m1; and DKK-1 assay id Hs00183740_m1. TaqMan One Step RT-PCR Master Mix Reagent (Life Technologies, Carlsbad, CA, USA) was used. The PCR reactions were performed using an ABI7500 Real-Time PCR system following the manufacturer’s protocol. The resulting data were analyzed using ABI software (R&D systems, Minneapolis, MN, USA).

### 2.6. Immunocytochemistry

DPCs (2 × 10^4^ cells per well) were seeded in 96-well plates and cultured overnight. After washing with PBS, DPCs were fixed with 4% paraformaldehyde at room temperature for 10 min. Cells were then permeabilized with PBS containing 0.1% triton × −100 and blocked with PBS containing 5% FBS and 1% BSA. After consecutive incubation with primary antibodies (200:1 dilution, Abcam, Cambridge, UK) at 4 °C for 12 h and Alexa 488 nm or Alexa 594 nm conjugated secondary antibodies (1000:1 dilution, Thermo Fisher scientific, Waltham, MA, USA) at room temperature for 1 h, nuclei were stained with DAPI (2000:1 dilution, Thermo Fisher scientific, Waltham, MA, USA) in the dark for 10 min. High-resolution fluorescence images were taken using an EVOS^TM^ FL Auto2 Imaging System (Thermo Fisher scientific, Waltham, MA, USA).

### 2.7. Data and Statistical Analysis

All experimental data are presented as the mean ± standard deviation (S.D.) of at least five independent experiments, unless otherwise indicated. The statistical significance of the difference was determined using Student’s *t*-test. A value of *p* < 0.05 was considered statistically significant. 

## 3. Results

### 3.1. Escin Activated Wnt/β-Catenin Signaling in WRHEK293A Cells 

**Figure 2:**
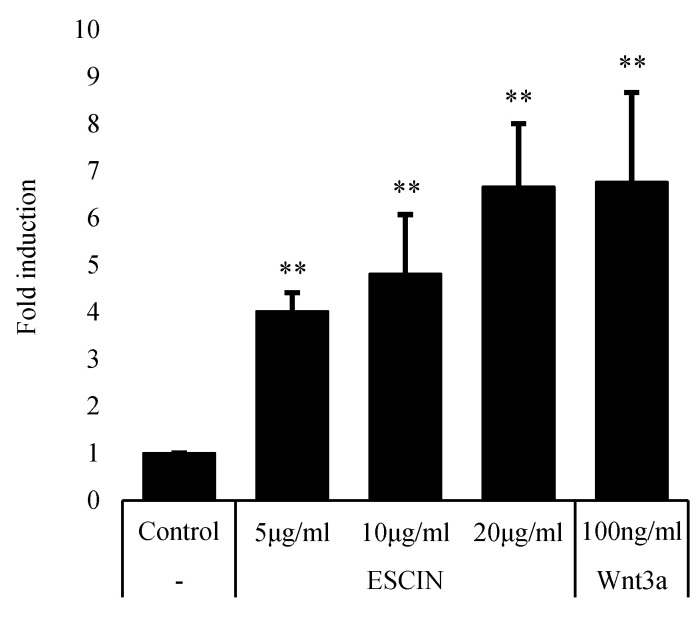
Escin activated Wnt/β-catenin signaling in HEK293 Wnt (TCF/LEF) reporter cells. Cells were treated with escin or recombinant Wnt3a for 24 h, lysed with 1× passive lysis buffer. GFP (488 nm/507 nm) was measured before luminescence measurement. Luciferase activity was measured by adding luciferase substrate. Luciferase activity, which refers to TCF/LEF transcriptional activity, was normalized to GFP signal, which is constitutively expressed in live cells. ** *p* < 0.01 compared to non-treated control (n = 5). Data are expressed as mean ± SEM.

We found that escin, a natural compound, stimulated Wnt signaling in Wnt reporter HEK293 cells. As shown in Figure 2, escin significantly increased the TOPFlash activity in a concentration-dependent manner. Recombinant Wnt3a, a biological Wnt ligand used as a positive control, also stimulated luciferase activity (Figure 2).

### 3.2. Escin Activated Wnt/β-Catenin Signaling in Cultured hDPCs

**Figure 3:**
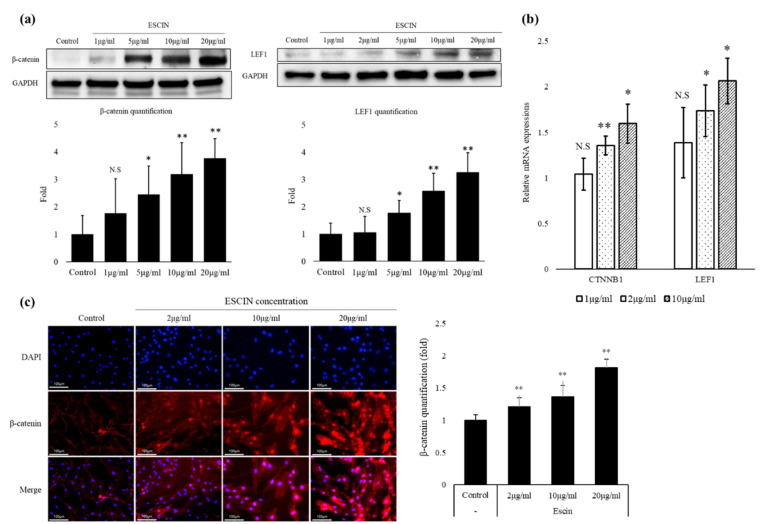
Escin activated Wnt/β-catenin signaling in cultured hDPCs. Cells were treated with various concentrations of escin for 24 h. (**a**) The protein expression levels of β-catenin and LEF1 were evaluated via Western blot analysis (n = 5). N.S: not significant, * *p* < 0.05, ** *p* < 0.01 compared to the non-treated control. (**b**) The mRNA expression levels of β-catenin and LEF1 (n = 5) were monitored via real-time PCR. N.S: not significant, * *p* < 0.05, ** *p* < 0.01 compared to the non-treated control. (**c**) A representative picture of the translocation of β-catenin from the cytosol to the nucleus, visualized via immunocytochemistry (n = 5). Data are expressed as mean ± SD.

To ensure the Wnt-stimulating activity of escin in normal cells, the effects of escin on the Wnt/β-catenin signaling in cultured hDPCs were investigated. The treatment of escin significantly increased the amount of cellular β-catenin protein, an indicator of Wnt signal activation, in a concentration-dependent manner (Figure 3a). The mRNA level of β-catenin was slight, but with statistical significance, and was increased by escin treatment in a concentration-dependent manner (Figure 3b). The protein and mRNA levels of the LEF1 gene, a cotranscription factor for β-catenin-directed gene expression, were also markedly increased by escin in a concentration-dependent manner (Figure 3a,b). The translocation of β-catenin into nuclei is one of the key processes of Wnt/β-catenin signal transduction. So, the nuclear localization of β-catenin in hDPCs after escin treatment was assessed via immunocytochemistry. As shown in Figure 3c, escin elicited the nuclear accumulation of β-catenin in a concentration-dependent manner, visually confirming that escin is a canonical Wnt agonist.

### 3.3. Escin Stimulated the Expression of Wnt Target Genes and Wnt Receptors

**Figure 4:**
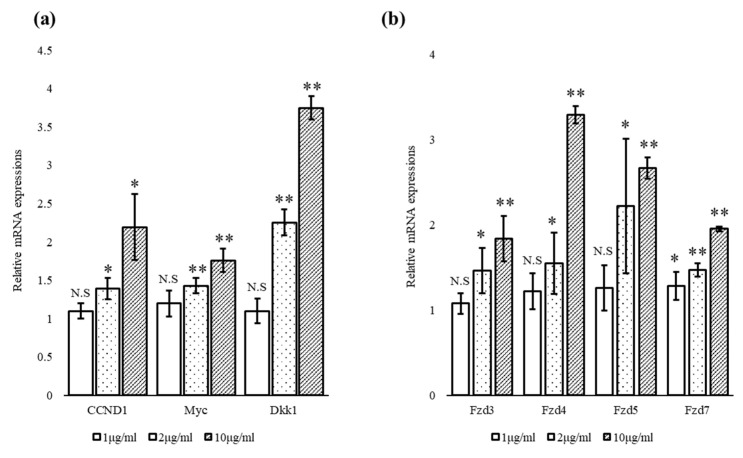
Escin stimulated the mRNA expression of Wnt target genes and Wnt receptors. (**a**) The mRNA expression of CCND1, Myc and Dkk1 was evaluated in hDPCs treated with escin for 24 h and (**b**) the mRNA expression of Wnt receptors, including Fzd3, Fzd4, Fzd5 and Fzd7, was evaluated in hDPCs treated with escin for 24 h (n = 5). N.S: not significant, * *p* < 0.05, ** *p* < 0.01 compared to the non-treated control. Data are expressed as mean ± SD.

The effects of escin on the mRNA expression of several Wnt target genes and receptors in hDPCs were investigated. As shown in Figure 4a, 24 h treatment with escin significantly increased the expression of Wnt target genes such as cyclin D (CCND)-1, Myc and DKK-1 in a concentration-dependent manner. In addition, escin increased the expression of the frizzled (Fzd) Wnt receptors Fzd3, Fzd4, Fzd5 and Fzd7, up to five-fold (Figure 4b).

### 3.4. Escin Facilitated the Degradation of GSK-3β via 26S Proteasome

To elucidate the mechanism by which escin stimulates Wnt/β-catenin signaling, we scrutinized the protein levels of GSK-3β and Axin2, components of the β-catenin destruction complex.

**Figure 5:**
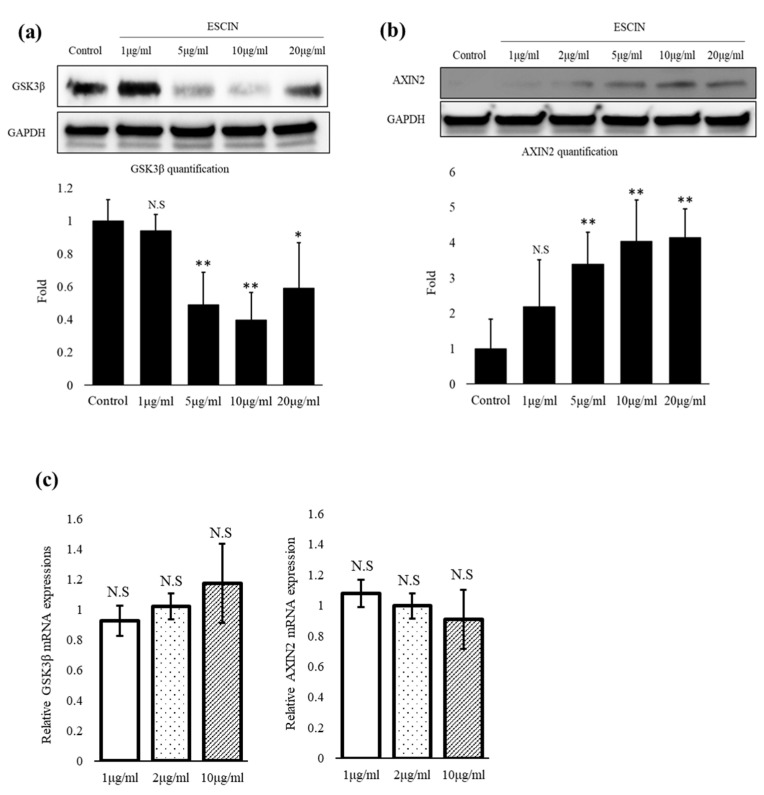

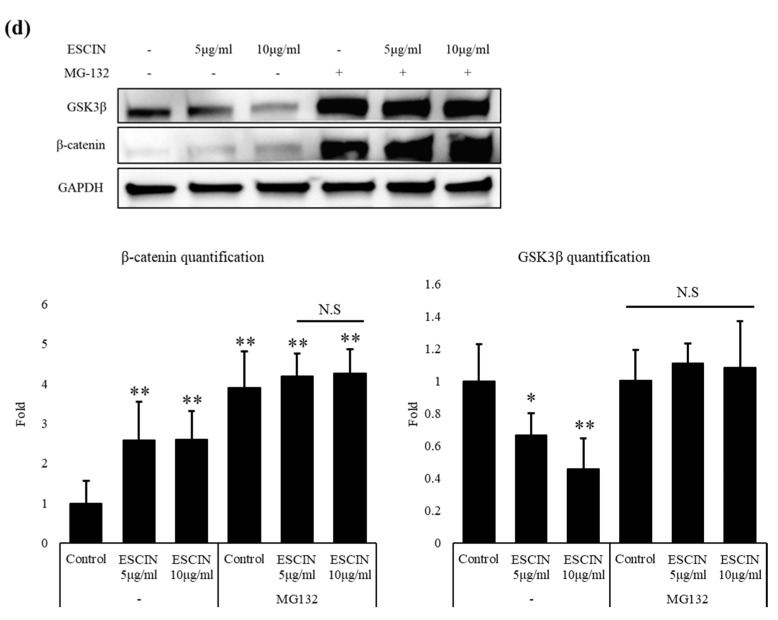
Escin facilitated the proteasomal degradation of GSK3-β in cultured hDPCs. The cells were treated with various concentrations of escin for 24 h. The expression levels of (**a**) GSK3 and (**b**) Axin2 proteins were evaluated via Western blot analysis (n = 5). N.S: not significant, * *p* < 0.05, ** *p* < 0.01 compared to the non-treated control. (**c**) The mRNA expression levels of GSK3 and AXIN2 were monitored via real-time PCR analysis (n = 5). N.S: not significant. (**d**) The effect of the proteasome inhibitor MG132 on the protein levels of GSK3 and β-catenin in hDPCs treated with escin for 24 h (n = 5). N.S: not significant, * *p* < 0.05, ** *p* < 0.01 compared to non-treated control. Data are expressed as mean ± SD.

Of note, escin drastically decreased the amount of GSK-3β protein in a concentration-dependent manner (Figure 5a). The protein level of Axin, on the other hand, was significantly increased by escin treatment in a concentration-dependent manner (Figure 5b).

There were no significant changes, however, in the mRNA levels of GSK-3 and Axin (Figure 5c). To determine whether escin led to the proteasomal degradation of GSK-3, we investigated the effect of MG132, a relatively specific 26S proteasome inhibitor, on the protein level of GSK-3. As shown in Figure 5d, the GSK-3 protein level depleted by escin was almost completely recovered in the presence of 10 µM MG132, indicating that escin facilitated the proteasomal degradation of the GSK-3 protein (Figure 5d). Furthermore, MG132 also dramatically increased the β-catenin protein level, and is reported to be another target of 26S proteasome in the Wnt/β-catenin pathway (Figure 5d).

### 3.5. Escin-Induced TOPFlash Activities Were Abrogated by sFRP1 and sFRP2 Treatment 

**Figure 6:**
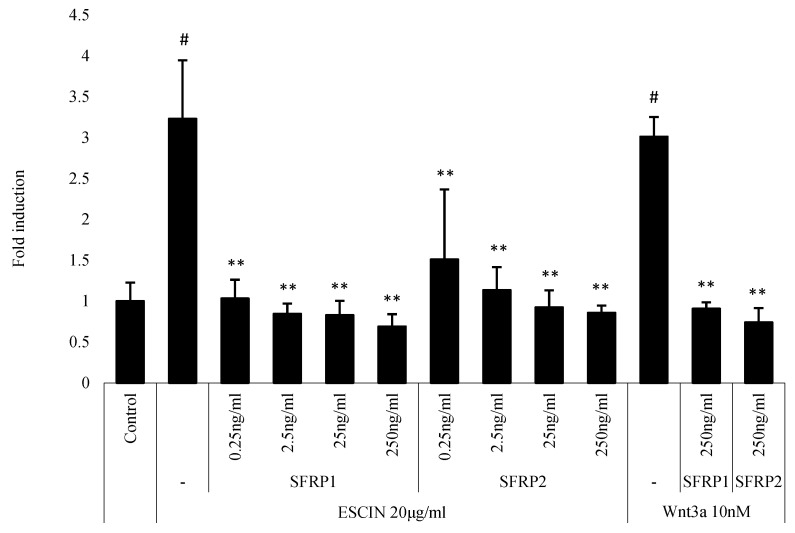
Escin-induced TOPFlash activity was abrogated by sFRP1 and sFRP2 treatment. WRHEK293A Wnt reporter cells were treated with escin in the presence of sFRP1or sFRP2 for 24 h. The cells were lysed, and GFP expression and TOPFlash activity were measured. Escin-induced TOPFlash activity was markedly abrogated by sFRP1 or sFRP2. Data are expressed as mean ± SD (n = 5). # *p* < 0.05 compared to the non-treated control. ** *p* < 0.01 compared to escin-treated control.

The secreted frizzled related proteins (sFRPs) were reported to inhibit Wnt signaling by interfering with the binding of Wnt ligands with their receptors, the frizzled receptors. To elucidate whether escin stimulates Wnt signaling through direct interaction with frizzled receptors, the effect of escin on Wnt reporter activity was evaluated in the presence of sFRP1 and sFRP2 in WRHEK293A reporter cells. As shown in Figure 6, escin-induced TOPFlash activities were significantly abrogated by sFRP1 and sFRP2 treatment in a concentration-dependent manner. The stimulatory activity of Wnt3a was also blocked by SFRP1/2 co-treatment. The treatment of sFRP1 or sFRP2 alone did not change Wnt reporter activity.

## 4. Discussion

**Figure 7:**
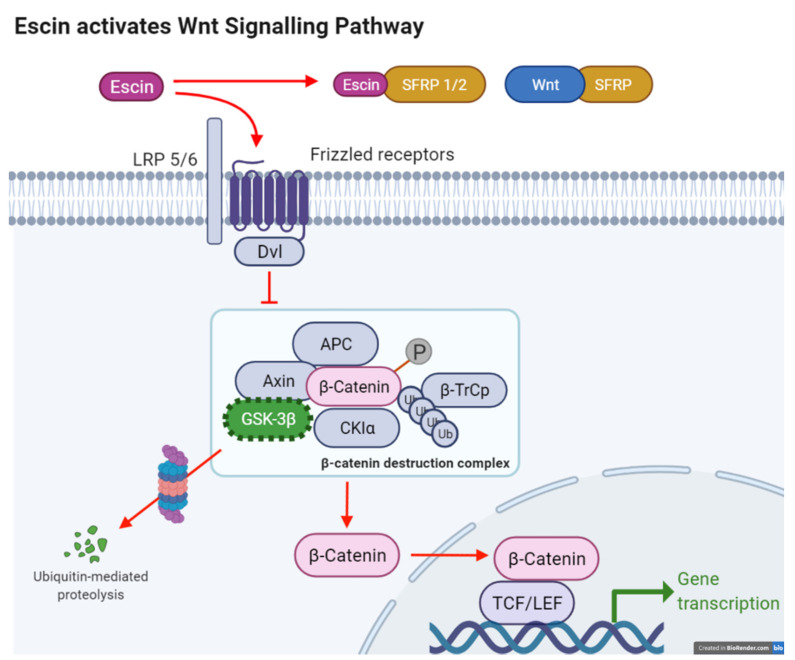
Summarized in vitro effects of escin on Wnt/β-catenin signaling pathway of human dermal papilla cells. All these effects in combination could lead to hair growth promotion.

In this report, we provide evidence, for the first time, to our knowledge, that the natural compound escin is an activator of the canonical Wnt/β-catenin signaling pathway (Figure 7). Wnt-stimulating activity was demonstrated in stable HEK293 Wnt reporter cells and in cultured hDPCs. Although a plethora of Wnt modulators have been developed and extensively reviewed in the literature, these are predominantly inhibitors because of their involvement in the pathogenesis of cancers [20,21,22,23,24]. As the abnormal inactivation of the Wnt/β-catenin signaling pathway has been reported to be associated with the pathogenesis of skin-related diseases like androgenetic hair loss, premature hair greying (canities) and vitiligo, Wnt activators could be a therapeutic option for these diseases [15,25,26,27,28,29]. Despite the therapeutic potential of Wnt signaling activators, few small-molecule Wnt activators have been demonstrated. 

In the present study, we found that escin remarkably stimulated Wnt/β-catenin signaling in stable TOPFlash reporter cells (Figure 2). In order to verify the Wnt-stimulating activity of escin in normal cells, the effects of escin on Wnt signaling in cultured hDPCs were investigated. As expected, the amount of β-catenin protein was increased by escin treatment in cultured hDPCs, suggesting that escin acted as a canonical Wnt agonist (Figure 3). The accumulation of β-catenin in the nucleus, a key process in transducing signals, was visualized via immunocytochemistry. The protein level of LEF1, a cotranscription factor that binds to β-catenin, was also increased by escin treatment. As shown in Figure 4, escin stimulated the mRNA expression of several genes, such as CCND1, Myc and DKK-1, which were reported to be upregulated by Wnt, confirming its role as a Wnt activator [30,31,32,33]. Our data strongly suggest that escin is a novel agonist of the canonical Wnt/β-catenin signaling pathway. In particular, because canonical the Wnt/β-catenin signaling pathway is continuously reported as a key factor in hair follicle regeneration and anagen elongation, escin could be considered an alternative pharmacological solution for hair loss. 

The β-catenin destruction complex, consisting of Axin, adenomatous polyposis coli (APC), casein kinase (CK)-1 and GSK-3β, is reported to play a crucial role in the pathway by tightly controlling the level of nuclear, transcriptionally active β-catenin [34,35]. In this context, we scrutinized the effects of escin on the components of the β-catenin destruction complex, especially on the protein levels of Axin and GSK-3. We found that the protein level of GSK-3β was significantly decreased by escin, suggesting that the depletion of the GSK-3 enzyme possibly resulted in the removal and/or dissociation of the β-catenin destruction complex, followed by β-catenin accumulation. GSK-3β plays a pivotal role in controlling Wnt signaling through the phosphorylation of β-catenin. The phosphorylated β-catenin is then subjected to ubiquitination-mediated proteasomal degradation, silencing Wnt signaling [36]. It is noteworthy that many of the Wnt activators reported to date are inhibitors of GSK-3β enzyme activity, preventing the phosphorylation of β-catenin and subsequent proteasomal degradation [37]. We also found that the treatment of MG132, a proteasome inhibitor, fully restored the protein level of GSK-3β, which was downregulated by escin (Figure 5d). Our data strongly demonstrate that escin activates Wnt signaling through the downregulation of the GSK-3β protein by promoting proteasomal degradation. This is consistent with previously reported articles demonstrating that the 26S proteasome is responsible for protein degradation in the Wnt signaling pathway [4]. The precise mechanisms by which escin facilitates the degradation of GSK-3, however, remain to be explored. 

Another control point of the Wnt signaling pathway is the scaffold protein Axin, which is thought to be the concentration-limiting component of the β-catenin destruction complex [38,39,40]. There are two isoforms of Axin proteins, Axin1 and Axin2, and the regulation of Wnt signaling by Axin proteins is complicated. The protein levels of both Axin1 and Axin2 are controlled by tankyrase. The inhibition of tankyrase increased the protein levels of both Axins, leading to stabilization of the destruction complex and the inhibition of Wnt signaling [38]. A recent study reported that Axin2, rather than Axin 1, was required for the formation of the β-catenin degradosome in SW480 colorectal cancer cells treated with a tankyrase inhibitor [41]. In the absence of tankyrase inhibitors, however, it seems different. When there is no Wnt signal, Axin is continuously degraded by poly(ADP-ribosyl)ation (PARylation)-dependent ubiquitination to limit β-catenin destruction complex formation, ensuring that the cells are receptive to Wnt stimuli and maintaining low Axin protein level constitutively. Upon Wnt stimulation, PARylated Axin is stabilized and the interaction with the low-density lipoprotein receptor-related protein (LRP)5/6 is facilitated, forming the Wnt signalosome [42,43]. Taken together, the role and behavior of Axin proteins seem context-dependent, depending on the presence of Wnt activators and/or other effectors like tankyrase inhibitors. In the absence of tankyrase inhibitors, the baseline level of Axin was very low, barely detectable by Western blot, but significantly increased upon Wnt activation by escin, demonstrating that the Axin protein had been stabilized (Figure 4b). It remains to be clarified whether the Axin protein occurred via signalosome formation or whether there were other mechanisms. The increase in the Axin2 protein, however, seems to be a consequence of Wnt activation, but not the cause, as there were no effectors of Axin proteins in the experimental system.

Our data demonstrate that the activation of Wnt signaling by escin resulted from the post-translational modifications of GSK-3 and AXIN since escin did not elicit any changes in mRNA levels (Figure 5c), which is consistent with previously reported literature where the Wnt signaling pathway is regulated by a wide range of post-translational modifications [44,45].

To investigate the possible interactions between escin and Wnt receptors, Wnt reporter cells were treated with secreted frizzled-related proteins (sFRPs) in the presence of escin. sFRPs are known to inhibit Wnt signaling by binding to Wnt ligands, thus depleting free, active Wnt ligands. Each sFRP (sFRP1, sFRP2, sFRP3, sFRP4 and sFRP5), however, was reported to show a different mode of action when modulating Wnt/β-catenin signaling, depending on the concentration balance of the Wnt and sFRP proteins. We found that the treatment of sFRP1 and sFRP2 almost completely blocked escin-induced Wnt signal activation, implying a possible direct interaction between escin and frizzled receptors (Figure 6). The precise interactions between escin, frizzled receptors, LRPs and sFRPs remain to be elucidated.

## 5. Conclusions

Our findings demonstrate that the natural compound escin stimulates canonical Wnt/β-catenin signaling through the downregulation of GSK-3β protein expression by facilitating the proteasomal degradation of GSK-3β. Our data strongly demonstrate the possible therapeutic potential of escin for treating and/or preventing Wnt-related disorders, especially skin-associated ones such as hair loss (androgenetic alopecia, female-pattern hair loss), canities and vitiligo, without safety concerns, since escin has long been used for dermatologic and cosmetic purposes.

## Data Availability

The datasets used and/or analyzed during the current study are available from the corresponding author on reasonable request. Some data may not be available because of the policy of the company and ethical restrictions.
